# Prevalence and risk factors of high cholesterol and triglycerides among people with HIV in Texas

**DOI:** 10.1186/s12981-022-00467-y

**Published:** 2022-09-19

**Authors:** Justin Buendia, Sabeena Sears, Osaro Mgbere

**Affiliations:** 1 Texas Department of State Health Services, Austin, TX USA; 2 Disease Prevention and Control Division, Houston Health Department, Houston, TX 77054 USA

**Keywords:** HIV, Cholesterol, Triglycerides, Medical Monitoring Project, Texas

## Abstract

**Background:**

People with HIV (PWH) commonly have elevated cholesterol and triglycerides levels that have been linked to medications. However, healthy behaviors including lifestyle changes can lower high cholesterol (CHOL) or high triglycerides (TG), thereby reducing individual risk for cardiovascular diseases. This study aimed to determine the prevalence and risk factors associated with high CHOL or TG among PWH in Texas.

**Methods:**

Cross-sectional data of 981 PWH from the 2015–2017 Texas and Houston Medical Monitoring Projects were examined. High CHOL or TG was identified by medical chart diagnosis, CHOL or TG medication use, or most recent fasting lab ≥ 200 mg/dl (total CHOL) or ≥ 150 mg/dl (TG). High CHOL or TG associations with sociodemographic and clinical characteristics were assessed using Rao-Scott chi-square tests. Prevalence of high CHOL or TG development was calculated using multivariable logistic regression model.

**Results:**

High CHOL or TG prevalence was 41% with participants being mostly male (73%), ≥ 40 years (68%), with overweight (31%) or obesity (28%), and virally suppressed (62%). Compared with PWH < 40 years of age, PWH in their 40s, 50s, and ≥ 60s were 57%, 64%, and 62% more likely to have high CHOL or TG, respectively. Participants with overweight and obesity were 41% and 30% more likely to have high CHOL or TG than those with normal weight (BMI: 18.5– < 25), respectively.

**Conclusion:**

Since high CHOL and TG are modifiable CVD risk factors, increased education and lifestyle modification interventions are warranted to prevent the development of high CHOL or TG among PWH.

**Supplementary Information:**

The online version contains supplementary material available at 10.1186/s12981-022-00467-y.

## Introduction

The breakthrough and implementation of antiretroviral therapy (ART) in the 1990s has significantly improved the life expectancies of people with HIV (PWH) [1]. These have occurred through reduction in AIDS-associated comorbidities among PWH, leading to similar lifespans as those without HIV [2]. Cardiovascular disease (CVD) is the leading cause of death of Americans [3] and its associated comorbidities such as high cholesterol (CHOL) and elevated triglycerides (TG), contribute to disease burden among PWH [4]. Several systematic reviews have concluded that with PWH living longer, HIV might contribute to CVD and its associated comorbidities such as high CHOL, hypertension (HTN), and type II diabetes mellitus (DM) [5–7]. A meta-analysis by Shah et al*.* found that among 80 longitudinal studies, PWH were twice as likely to develop CVD compared to those without HIV [6]. This highlights the growing public health burden of CVD among PWH.

High CHOL and high TG are two of the most common contributors of morbidity and mortality among US adults. According to the 2017 National Center of Health Statistic data brief, approximately 1 in 8 Americans over the age of 20 had high serum total CHOL and about 1 in 5 had low levels of high-density lipoprotein (HDL) cholesterol [8]. High HDL has been shown to be associated with reduced CVD morbidity [9] and conversely, high levels of low-density lipoprotein (LDL) cholesterol has been linked to be one of the main drivers of CVD, especially myocardial infarctions and peripheral artery disease [10]. This is primarily due to LDL’s pro-inflammatory effects on macrophages and endothelial activation, which can lead to plaque buildup and eventual rupture [11]. Additionally, high circulating levels of TG has been associated with increased CVD risk mainly in part of their cholesterol-enriched remnant-like particles, which undergo microphage incorporation and entry to through the arteries to promote atherosclerosis [12]. Not only have both CHOL and TG been established as major independent risk factors for CVD [13], but it also adds to the already substantial burden of CVD on healthcare costs. According to the American Heart Association’s projections, direct and indirect costs of CVD totaled $555 billion in 2016 and is estimated to increase to $1.1 trillion by 2035 [14].

Compared with those without HIV, risk factors for high CHOL or TG are similar among PWH [15, 16]. It has been consistently shown that certain cohorts, such as those who have overweight (body mass index, BMI: 25–< 30 kg/m^2^) or obesity (BMI ≥ 30 kg/m^2^), have a family history of high CHOL or TG, age ≥ 40 years, smokers, and African Americans have relatively higher odds of developing high CHOL or TG [15, 16]. Among PWH, additional risk factors such as ART adherence, length of HIV duration, and certain classes of ART therapies have been linked with impaired lipid metabolism [5, 17, 18].

ART medications, particularly those that utilize protease inhibitors, have been shown to be linked with negative effects on serum total CHOL and TG [19–22]. Considering the development of more recent ART therapies that have not been associated with such cardiometabolic abnormalities, earlier generations of ART medications such as indinavir and stavudine, have been prescribed less frequently [23]. With this potentially negative side effect of first-generation ART therapies, concerns have been raised that PWH, especially those who have been living longer with HIV and were taking first-generation ART medications, have a higher risk of developing atherosclerosis. A recent systematic review among PWH observed that the prevalence of hyperlipidemia among PWH were between 28 and 80%, with high TG being the most common type of lipid abnormality [24]. The high variability of hyperlipidemia prevalence among PWH can be attributed to inherent differences between the study cohorts associated with geographical location, dietary and physical activity lifestyle, duration of time since HIV diagnosis, and variations in defining high CHOL and/or TG [24].

In the US, Southern states are disproportionally affected by both HIV and CVD. In 2016, the greatest burden of CVD in the US was centered among Southern states, with Mississippi having the highest rate of CVD DALYs (Disability-Adjusted Life-Years), and Arkansas, Oklahoma, Louisiana, and Alabama rounding out the top 5 [25]. Regarding HIV prevalence by region, Southern states account for nearly half (44%) of all PWH in the country [26], while only representing 37% of the US population [27]. With the additive disproportionate burden of both CVD and HIV among PWH in the South, it is imperative to elucidate risk factors for both chronic disease, which, may lead to better risk mitigation and prevention practices. With the lack of data among PWH in the southern US, we aim to estimate the prevalence of high CHOL or TG and establish its associated risk factors among PWH in Texas.

## Methods

The Medical Monitoring Project (MMP) is an ongoing Centers for Disease Control (CDC) surveillance system that collects and assesses behavioral and clinical information among PWH in 23 project areas from various cities, states and territories in the US. For these analyses, medical record abstraction and interview data obtained from the 2015–2017 cycles of the Texas and Houston MMP project areas were utilized. Data collection was based on a two-stage sampling method with the first stage at the state/project area level and the second stage consisting of a simple random sample of persons diagnosed with HIV aged 18 years and older that was drawn for each project area from the National HIV Surveillance System (NHSS) in which each participant had an equal probability for selection.

Data collection occurred between June 2015 and May 2018, and data were weighted by CDC on the basis of known probabilities of selection at state or territory and person levels and non-response [28] and post-stratified to NHSS population totals [29]. Our final analytic sample included 981 PWH who participated Texas and Houston MMP. Behavioral and clinical data from the study participants were collected using an in-person and telephone interview as well as medical record abstraction. High CHOL and high TG were identified by formal diagnosis recorded in the medical chart, prescription of CHOL or TG-lowering medications, or most recent fasting lab of ≥ 200 mg/dl (total CHOL) or ≥ 150 mg/dl (TG).

Sociodemographic variables included age, sex at birth, race/ethnicity, education level, poverty level, height, weight, insurance, binge drinking, smoking status, unmet need, any non-injection or injection drug use, and diagnoses of HTN and DM. BMI was calculated by dividing the most recent weight (in kg) by the most recent height (in meters^2^) from abstracted medical records within 2 years prior to interview.

Insurance coverage was assessed by asking participants about all the types of insurance they had in the last 12 months as well as their historical and current smoking status and frequency. Poverty was assessed from household income, the number of people depending on the income, and the federal poverty line for the year that the patient was interviewed. Binge drinking was defined by ≥ 5 drinks for men or ≥ 4 drinks for women over the course of one sitting [30] in the last 30 days. Similar to how high CHOL or TG was determined, DM and HTN were determined by either a formal medical chart diagnosis, prescription of insulin and glucose control medications for DM or anti-hypertensive medications for HTN, and a most recent fasting blood glucose level of > 126 mg/dl (DM) or an average of the last three systolic/diastolic blood pressure readings of 140/90 mmHg (HTN) [31, 32]. Major or other depression was defined as a score of 10 or more on the Patient Health Questionnaire depression scale (PHQ-8) [33]. Unmet need was assessed with a 19-question needs assessment regarding services (Additional file 1) that were needed but not received within the past 12 months. HIV-related clinical variables included: years of HIV diagnosis, geometric mean CD4^+^ T-lymphocyte (CD4) count and durable viral load. A participant was classified to have a sustained undetectable viral load if all the viral loads for 12 months prior to the interview were undetectable (≤ 200 copies/μl).

### Statistical analysis

Weighted prevalence and 95% confidence intervals (CI) of high CHOL or TG were calculated as an overall measure and by each of the following categories of sociodemographic and HIV-related characteristics: age group, sex at birth, race/ethnicity, education, poverty, insurance, BMI, smoking status, binge drinking in the past 30 days, number of unmet needs, DM status, HTN status, depression, any drug use, time since HIV diagnosis, mean CD4 count and sustained viral load. A composite unmet need score was calculated by scoring participants based on how many services were needed but not received. The sum of all unmet needs was then classified into three levels of unmet need (0, 1, and ≥ 2).

Associations between sociodemographic characteristics among participants and high CHOL or high TG status were assessed using Rao-Scott chi-square tests with significance threshold *p* < 0.05. To identify factors associated with high CHOL or TG and to estimate adjusted prevalence ratios (aPR) and corresponding 95% CIs, multivariable logistic regression model analyses were conducted with high CHOL or TG as the outcome. Only variables that retained statistical significance in the univariable models with *p* < 0.10 as well as those that are epidemiologically significant were included as independent predictors in the multivariable model. Although HIV diagnosis duration was significant in the univariable models, it was dropped in the multivariable model because of its moderate correlation with age (ρ = 0.47). All analyses accounted for complex sample design and unequal selection probabilities and were conducted using SAS 9.4 (SAS Institute Inc., Cary, NC, USA) and SAS-callable SUDAAN 11.0 (RTI International, Research Triangle Park, NC, USA).

## Results

Table 1 illustrates the baseline characteristics of these participants by high CHOL or high TG status. Out of the 981 total MMP participants in our analysis, 41% had high CHOL or high TG (n = 400), most of whom were men (76%), non-Hispanic Black (35%) or Hispanic (34%), ≥ 50 years of age (55%), had overweight (40%) or obesity (36%), had ≥ 1 unmet need (49%), had HTN (83%), two-thirds were living with HIV for ≥ 10 years, and 74% had undetectable sustained viral loads. Significant differences were observed between race/ethnicity, age group, BMI, insurance, unmet need, depression, smoking status, any drug use in the past 12 months, DM, HTN, HIV diagnosis duration, mean CD4 count, and durable viral load. (*p* < 0.05 for all).

**Table 1:** Baseline characteristics by high cholesterol or triglycerides status of people with HIV in Texas

Characteristic	High Cholesterol/Triglycerides Status						Test Statistics	
	Normal CHOL or TG		High CHOL or TG		Total			
Sex	N	%^§^	N	%^§^	N	%^§^	Rao-Scott Chi-square value	p-value
Male	412	58	302	42	714	100	2.23	0.13 ns
Female	169	65	98	35	267	100		
Race/Ethnicity								
White	106	53	103	47	209	100	12.77	< 0.01**
Black	293	69	140	31	433	100		
Hispanic	147	55	137	45	284	100		
Other	35	57	20	43	55	100		
Age group (years)								
18–39	250	80	64	20	314	100	63.97	< 0.001***
40–49	152	58	115	42	267	100		
50–59	125	47	145	53	270	100		
≥ 60	54	42	76	58	130	100		
BMI (kg/m^2^)								
18.5– < 25 (normal)	219	70	89	30	308	100	26.88	< 0.001***
25–< 30 (overweight)	149	48	159	52	308	100		
≥ 30 (obesity)	134	49	142	51	276	100		
Education								
< High school	96	55	82	45	178	100	3.97	0.14 ns
High school/equivalent	170	65	98	35	268	100		
> High school	314	59	220	41	534	100		
Insurance								
Private	207	59	153	41	360	100	8.25	0.041*
Public	243	56	180	44	423	100		
Ryan White only	94	66	57	34	151	100		
Unspecified/None	32	79	9	21	41	100		
Poverty								
Above	302	58	227	42	529	100	0.30	0.58 ns
At or below	239	60	154	40	393	100		
Unmet need								
0	217	52	206	48	423	100	14.72	< 0.001***
1	125	63	78	37	203	100		
≥ 2	238	67	116	33	354	100		
Smoking status								
Never	285	61	198	39	483	100	28.83	< 0.001***
Former	76	41	104	59	180	100		
Current	215	69	98	31	313	100		
Binge drinking								
No	473	60	324	40	797	100	< 0.01	0.96 ns
Yes	104	60	74	40	178	100		
Any Drug Use								
No	413	56	327	44	740	100	14.67	< 0.001***
Yes	162	72	73	28	235	100		
Diabetes								
No	540	67	285	33	825	100	86.28	< 0.001***
Yes	37	23	112	77	149	100		
Hypertension								
No	181	73	68	27	249	100	36.92	< 0.001***
Yes	345	51	332	49	677	100		
Depression								
No	437	58	317	42	754	100	4.98	0.026*
Yes	140	68	79	32	219	100		
HIV related characteristics								
HIV diagnosis duration								
< 5 years	161	74	60	26	221	100	23.96	< 0.001***
5–9 years	139	66	72	34	211	100		
≥ 10 years	281	52	268	48	549	100		
Mean CD4 count (cells/µl)								
0–199	54	71	24	29	78	100	10.21	0.017 *
200–349	59	59	40	41	99	100		
350–499	79	57	59	43	148	100		
≥ 500	255	50	243	50	498	100		
Durable viral load (copies/ml)								
< 200 (undetectable)	316	52	296	48	612	100	29.81	< 0.001***
≥ 200	265	72	104	28	369	100		

^§^Within a given level of the characteristic, some row percentages may not add up to exactly 100 due to rounding; Significance Level: *p < 0.05, **p < 0.01, ***p < 0.001; ns: Not Significant (p > 0.05)

Table 2 highlights the aPRs and their corresponding 95% CIs of having high CHOL or high TG by the associated variables in the multivariable model. Among PWH in Texas, age, BMI, and having DM were the main variables associated with having high CHOL or high TG. Those in their 40s, 50s, and ≥ 60, had a 57%, 64% and 62% increased prevalence of having high CHOL or TG, respectively, compared to those 18–39 years of age. Compared to those with normal weight (BMI: 18.5– < 25 kg/m^2^), participant with overweight and obesity had 41% (95% CI 1.16–1.71) and 30% (95% CI 1.05–1.62) higher odds of having either high CHOL or high TG, respectively. Those who had DM had almost a twofold increased likelihood of having either high CHOL or TG (aPR = 1.91; 95% CI 1.65–2.22) compared to those who were not diabetic. Conversely, former smokers were 28% more likely to have either high CHOL or TG compared to never smokers. Although we observed significant associations between high CHOL or TG with birth sex, race/ethnicity, insurance, unmet need, depression, drug use, smoking status, HTN, mean CD4 count, and durable viral load, we did not observe similar significant prevalence of having high CHOL or TG in the multivariable model.

**Table 2:** Adjusted prevalence ratio of high cholesterol or triglycerides among people with HIV in Texas

Characteristic	aPR	95% CI
Birth sex		
Male *(Ref)*	1.00	–
Female	0.97	0.81–1.16^ns^
Race/Ethnicity		
White *(Ref)*	1.00	–
Black	0.81	0.65–1.01^ns^
Hispanic	1.11	0.90–1.36^ns^
Other	0.72	0.49–1.06^ns^
Age Group (years)		
18–39 *(Ref)*	1.00	–
40–49	1.57	1.20–2.05*
50–59	1.64	1.24–2.17*
≥ 60	1.62	1.17–2.25*
Insurance		
Private *(Ref)*	1.00	–
Public (No Ryan White)	0.99	0.83–1.17^ns^
Ryan White only	1.06	0.86–1.31^ns^
None/unspecified	0.61	0.34–1.11^ns^
Unmet need		
0 *(Ref)*	1.00	–
1	0.91	0.74–1.12^ns^
≥ 2	0.88	0.73–1.06^ns^
Depression		
None *(Ref)*	1.00	–
Major/other depression	0.82	0.67–1.01^ns^
Drug use		
None *(Ref)*	1.00	–
Any	1.06	0.90–1.27^ns^
BMI (kg/m^2^)		
< 25 *(Ref)*	1.00	–
25–< 30	1.41	1.16–1.71*
≥ 30	1.30	1.05–1.62*
Smoking status		
Never *(Ref)*	1.00	–
Former	1.28	1.05–1.56^*^
Current	0.99	0.81–1.21^ns^
Diabetes		
None *(Ref)*	1.00	–
Yes	1.91	1.65–2.22^*^
Hypertension		
None *(Ref)*	1.00	–
Yes	1.17	0.96–1.42^ ns^
Mean CD4 count (copies/µl)		
0–199	0.68	0.48–0.97^*^
200–349	0.95	0.74–1.20^ns^
350–499	0.94	0.76–1.15^ns^
≥ 500 *(Ref)*	1.00	–
Durable viral load (copies/ml)		
Undetectable (< 200) *(Ref)*	1.00	–
Detectable (≥ 200)	0.98	0.81–1.19^ns^

aPR: Adjusted Prevalence Ratio, 95%CI: 95% Confidence Interval, Ref: Referent

Significance Level: *Significance based on 95% confidence interval, ns: Not Significant (p>0.05)

## Discussion

With PWH living longer, it is imperative that we monitor modifiable risk factors such as high CHOL and high TG. Our results show that traditional CVD risk factors such as increased BMI and age, smoking, DM, and HTN were prevalent among our participants. Such risk factors have been consistently shown among PWH [34–37]. Although we found that former smokers were more likely to have high CHOL or TG, this may be explained by BMI. Former smokers had a mean BMI of 30 compared to never smokers (mean BMI = 28 kg/m^2^). We found that four in ten PWH in Texas have high CHOL or high TG. This is consistent with the Data Collection on Adverse Events of Anti-HIV Drugs (D:A:D) study, a prospective multi-national cohort of over 17,000 PWH in 188 clinics in 20 countries in the US, Australia, and Europe, where 27% and 40% of patients on ART were observed to have high CHOL and high TG, respectively [38]. Given the disproportionate effect of CVD and its related risk factors such as high CHOL in the South, we hypothesized the prevalence of high CHOL to be higher than the 2015–2016 National Health and Nutrition Examination Survey (NHANES) estimates of 12% [8]. Comparatively, our much higher prevalence of approximately 40% could be explained by the inherent demographic variations of our sample, such as a predominately older sample population, our study is among PWH while NHANES is among the general population, and that the NHANES estimate is national while our sample resides in Texas. Conversely, our findings are lower than higher range of estimates of previous studies on cholesterol among PWH [7, 24], and this may be partially because we only sample PWH in Texas, not the entire southern US.

Additionally, CHOL levels vary significantly with age, race/ethnicity, and birth sex. In a longitudinal cohort study that compared PWH by birth sex, low density lipoprotein (LDL) and HDL ratios was higher among women compared to men after the start of ART [39]. The authors also observed that E-selectin, a pro-inflammatory marker linked with increased atherosclerotic risk [40] and HIV-associated vascular inflammation [41], remained elevated among women than men. This finding, along with a Massachusetts-based case–control study [17] indicates that immunological processes are less effective among female PWH compared to male. Furthermore, a cross-sectional pharmacogenetic study observed that compared with White and Hispanic PWH, Black PWH had lower plasma TG levels, but the greatest increase in TG levels when exposed to protease inhibitors [42]. Our sample was comprised of predominantly men (73%), those aged ≥ 40 years (68%) and were either of non-Hispanic Black (44%) or Hispanic (29%) ethnicity, which were all been shown to be associated with higher prevalence of high CHOL [43]. Additionally, a study on rural women in North Carolina revealed that compared to Caucasian women, Black women had lower education and income, but a higher mean BMI and prevalence of HTN, DM, and angina [44], postulating that targeted risk reduction efforts are needed. Additionally, we used a more parsimonious diagnosing algorithm by which only those with marked fasting CHOL and TG levels were included in the analysis. For instance, participants who had unknown fasting lab status were excluded, thus the majority of those who had high CHOL or TG were from formal diagnosis or intake of CHOL or TG-lowering medications from their medical charts (97%). Another reason for the lower-than-expected prevalence compared to other studies on CHOL among PWH is that most of our participants (77%) have been living with HIV for over five years, making them more likely aware of the details of their HIV care and therefore may be in better overall general health.

There are currently no published regional estimates of high CHOL or high TG prevalence among PWH living in the Southern US, although estimates vary from 28 to 80% across various cohorts who are living with HIV [45–49]. One of the main contributors to the wide variation of PWH prevalence is the differences among study participants by known predictors of high CHOL or high TG. The evolution of ART medications combined with the inherent difference among the PWH cohorts explains this huge variability in CHOL and TG prevalence.

Historically, different ART classes have been found to have different effects on lipids among PWH. Generally, ART medications that contain protease inhibitors (PIs), non-nucleoside reverse transcriptase inhibitors (NNRTIs), and nucleoside reverse transcriptase inhibitor analogues (NRTIs) have been reported to adversely affect TG and LDL CHOL levels [24] through increased CHOL absorption [50]. The differential effects of various PI-containing ART regimens were observed in the Data Collection on Adverse Events of Anti-HIV Drugs (D:A:D) study, where they found ritonavir increased TG and LDL CHOL levels but saquinavir and nelfinavir had less adverse effects on cholesterol [51]. In a study using the same D:A:D cohort, cumulative dosage of several PI-containing ART medications was linked with increased risk of heart attacks [52]. With advances in modern HIV medicine, second-generation ART medications such as atazanavir, darunavir, maraviroc, dolutegravir and raltegravir were not observed to have adverse effects on the lipid profiles of PWH [53–55]. The evolution of ART medications combined with the common practice of combining certain classes of ART therapies together to create a comprehensive regimen makes it difficult to assess the holistic effect of ART regimens as each medication has different effects on overall lipid profiles among PWH.

Our multivariable logistic regression results show that none of the HIV-related variables were significantly associated with high CHOL or high TG prevalence. With the lack of temporal data on ART classes from MMP, our results align with previous studies that suggest that traditional risk factors among HIV-negative cohorts are the same risk factors for high CHOL or TG among PWH [56–58]. Several possible mechanisms have been proposed to explain why PWH have a higher prevalence of dyslipidemia compared to those without HIV. Chronic inflammation due to the HIV infection itself may increase chronic inflammation processes such as lipopolysaccharide formation [59] and also lower anti-inflammatory mechanisms such as reverse cholesterol transport [60] and HDL-cholesterol function [60, 61]. Since sustained viral load was found to have no association with high CHOL or high TG risk, this may suggest that our participants resemble the general population with similar risk factors such as comparable BMI [-49] and life expectancies [62]. However, because of the cross-sectional nature of MMP and the lack of data on when high CHOL or high TG was initially diagnosed and only looking at CD4/viral load data for the previous year, this was difficult for us to determine. We did find that those with the lowest mean CD4 count to have a lower likelihood of having high CHOL or high TG. This aligns with previous studies that showed an association between low CD4 count and CVD [63] and CHOL excretion [64] among PWH, as well as inflammation, and increased levels of activated CD4^+^ T cells, which are prominent in atherosclerotic lesions in the general population [65]. Systemic inflammation is also a byproduct of poor glucose regulation in T2DM [66], which could explain why we saw an increased prevalence of high CHOL or high TG among our participants with T2DM. With the rising public health burden of CVD and its related comorbidities [67], our study warrants increased awareness of such chronic diseases among PWH.

Our study addresses one of the HIV/AIDS Bureau performance measures on lipid screening [68]. According to the US Department of Health and Human Services, fasting lipid profiles should be monitored for PWH at the start of care, ART initiation or modification, and every 6 or 12 months depending on initial lipid levels [69]. With an aging cohort of PWH and given the disproportionate effect of both HIV and CVD among Southern PWH, it is imperative that CVD comorbidities such as CHOL and TG be regularly monitored as part of usual care among Southern PWH.

Our study had several notable strengths. A unique asset of MMP is its inherently robust sampling methodology, which is specifically designed to attain generalizability among PWH in Texas with the use of weighted sampling. Additionally, thorough medical chart abstractions provided a well-rounded representation of clinical data that allowed for the measurement of a wide range of demographic and cardiometabolic factors. Combined with detailed patient interviews that provided details on sociodemographic and other behavioral risk factors, we were able to capture a wide array of potential confounders on high CHOL or high TG among PWH, which enabled us to estimate independent associations of each of the significant predictors in our regression analysis.

However, our study is limited by the limited scope of MMP in terms of predicting CVD endpoints. MMP lacked details on other known predictors of high CHOL or TG such as familial history, physical activity, and diet [70]. Additionally, the majority of total CHOL and TG labs had unknown fasting status and therefore, could not be utilized for the current analysis. The majority (97%) of our high CHOL and TG cases were from formal medical chart diagnosis so the potential underestimating effect of not using unknown fasting labs to identify undiagnosed/untreated high CHOL or TG are minimal. Finally, an inherent limitation of cross-sectional cohort studies is the inability to deduce causality and the possibility of residual or uncontrolled confounding. We cannot infer if HIV itself was associated with a high prevalence of having high CHOL or high TG. We also did not have temporal data on types of ART medications. Future longitudinal studies where the specific length of duration of certain ART medications are known, it would be helpful to delineate the independent associations of specific ART classes on an aging cohort of PWH. CVD risk among PWH is a complex topic, which involves the interplay among lifestyle factors such as smoking, diet, and stress, other recognized CVD risk factors such as hypertension, and type II diabetes, and inflammation related to HIV and certain antiretrovirals. It is therefore imperative that PWH are aware of these risk factors and have their CHOL and TG levels checked regularly. PWH with elevated CHOL or TG would benefit from medical care through improved diet quality and nutrition as well as the use of statins and other lipid-lowering medications to help control their CHOL and TG levels.

## Conclusions

Our findings fill gaps in knowledge regarding high CHOL or high TG prevalence among PWH in Texas. To date, this study is the first to estimate high CHOL or high TG prevalence among PWH in Texas. These findings underscore the fact that traditional CHOL or TG risk factors among the general population also play paramount roles in dyslipidemia among PWH. This statewide assessment is crucial for prioritizing risk mitigation and primary care prevention services in an aging cohort of PWH. Due to PWH living longer with HIV and the subsequent rise HIV prevalence in recent years PWH have a higher risk of developing chronic diseases such as high CHOL or high TG, compared to the general population. Given that the lifespans of PWH are comparable to those without HIV, as well as increasing dyslipidemia prevalence in the general population, longitudinal studies are warranted to assess long-term risk of high CHOL or high TG and how it may impact mortality among PWH. Since HIV care providers are usually the primary care providers for PWH, it is important for them to regularly screen and monitor chronic disease risk factors among PWH, and to make timely referrals to specialists when appropriate. Moreover, intervention programs that promote overall healthy lifestyle such as increased physical activity, decreased sedentary behaviors, and nutritional education should be easily accessible to PWH as part of an integrated HIV care during scheduled clinic visits.

## Disclaimer

The findings and conclusions of this article are solely the responsibility of the authors and do not necessarily represent the official position of the US Centers for Disease Control and Prevention or Texas Departments of State Health Services or Houston Health Department.

## Supplementary Information

**Additional file 1.** Questions used to create unmet need composite score.

## Data Availability

The datasets generated and/or analyzed during the current study are not publicly available but may be made available upon reasonable request from the corresponding author subject to applicable policies of the organizations that the authors are affiliated.
