# Supplementary material for: Prevalence and risk factors of high cholesterol and triglycerides among people with HIV in Texas
Source: AIDS Res Ther. 2022 Sep 19;19:43. doi: 10.1186/s12981-022-00467-y (PMC9484232; doi:10.1186/s12981-022-00467-y)
Supplement: Supplementary file 1 — Additional file 1. Questions used to create unmet need composite score. [file 12981_2022_467_MOESM1_ESM.docx]

**Supplemental Table I: Questions used to create unmet need composite score**

| **Services needed but not received within the past 12 months** |
| --- |
| HIV Case Management Services?  Dental services?  Domestic violence services?  Personal food assistance?  HIV peer group support?  Interpreter services?  A lawyer or legal advice?  Mental health services?  Meals/food service?  Medicines though the AIDS Drug Assistance Program (ADAP)?  Patient navigator services?  Shelter services or housing service?  Public benefits including SSDI (social security disability insurance)?  Public benefits including SSI (social security insurance)?  Drug or alcohol counseling?  Adherence services?  Transportation assistance? |
